# Medical Opinion and Movement

**Published:** 1908-02-29

**Authors:** 


### Medical Opinion and Movement.

THE Ethical Committee of the British Medical Association, in view of the fact that the term "hospital" is sometimes improperly applied to buildings or institutions, have circulated amongst the divisions of the Association a definition which this committee has drawn up in order to prevent abuse of the word in future. The committee very properly hold that any institution to which the term "hospital" is applied must at least possess the following characteristics: "that it receives necessitous patients, and is maintained wholly or partially by public funds and charitable contributions; that the control, including the appointment of the staff, is in the hands of medical or lay persons appointed by, or responsible to, those who provide the funds; that the institution is not established or conducted for the pecuniary profit or personal gain of the body or persons who control it." We have not always been in agreement with the action taken by the British Medical Association, but we feel the course pursued by the Ethical Committee should receive the support of the profession. We hope, therefore, that the proposed definition may receive the endorsement of the representative meeting in the autumn.

---

ONE of the chief lessons which all medical men must learn in the course of their experience is to be careful and guarded in prognosis. It is important for their own reputation. A reputation is more easily lost than won by a hasty opinion. It is of still greater importance in the interests of their patients. Prognosis must always largely depend upon diagnosis, and in the present condition of medical knowledge a certain diagnosis is often impossible. No better object-lesson of these facts could be offered than the account by Mr. Mayo Robson of cases of abdominal tumours which, though apparently malignant, have proved otherwise by their disappearance and by the complete recovery of the patients. Among these are found cases of tumour of the lower bowel giving rise to obstruction in which laparotomy has confirmed the diagnosis of malignancy, a colotomy or short-circuiting operation has been performed, and the whole condition has subsequently cleared up entirely. Cases of gastric tumours are cited, in which all the symptoms suggestive of malignancy were present, but complete recovery has followed the operation of gastro-enterostomy. Finally Mr. Robson describes some remarkable cases of tumour involving the liver and gall bladder where removal or simple exploration have led to a permanent cure. Such cases go a long way to support the proposition that if a diagnosis be at all doubtful an exploratory operation should be performed. Even if nothing radical can be done, in certain cases at least the operation *per se* appears often to exercise some hitherto unexplained influence upon the morbid condition, and lead to recovery. A perusal of these cases demonstrates, too, the danger of forming a dogmatic opinion, and is a salutary lesson against hasty diagnosis and prognosis.

DECHLORIDATION is now a recognised principle of treatment in cases of Bright's disease. The failure of the kidneys to excrete chlorides leads to their accumulation in the tissues, which again causes the passage of lymph from the blood-vessels into the tissues and consequent œdema and anasarca. Martinet, in discussing the subject recently, points out the desirability of clearly distinguishing those conditions of renal trouble in which dechloridation should be carried out. When acute or chronic nephritis is accompanied by œdema of a greater or less degree, such dechloridation is clearly indicated. If, however, there is no œdema, the necessity for dechloridation must be determined by further examination for retention of chlorides and for retention of fluid in the tissues. With a normal diet the daily excretion of chlorides in the urine should amount to 10 or 12 grammes, so that an appreciable diminution from this amount may be taken as evidence of a retention of chlorides in the system. The second sign, still more characteristic of a tendency towards dropsy, consists in a notable and progressive increase in body weight. Such an increase is due to the retention of fluid in the tissues, and corresponds to the diminution in excreted chloride. It is estimated that one litre of water is retained for every six grammes of sodium chloride. It is only when this retention of fluid has attained a certain degree that œdema of the tissues becomes manifest. Treatment consists in regulating the ingestion of chlorides by suitable diet and in administering drugs calculated to augment the excretion of chlorides. The traditional milk and fruit diet for such cases is undoubtedly also the most suitable from this point of view. In such cases where an exclusively milk diet is not necessary or desirable the amount of salt per diem should be fixed and regulated. In so far as the retention of fluid depends upon the retained chlorides, restriction in the amount of fluid taken seems of secondary importance.

---

DR. H. C. MANN has recently carried out some extensive observations on the blood-pressure in man under varying conditions, both of health and disease, and his results are of considerable interest and importance. They clearly demonstrate that the manometer is an instrument of definite clinical significance, and can be utilised as a useful guide to pathological conditions for those who learn to interpret it aright. In healthy individuals the difference between systolic and diastolic readings measures about 30 millimetres of mercury. Vigorous muscular exercise in the "untrained" is accompanied by a rise in the systolic arterial pressure, with high irregular readings, the diastolic pressure remaining low, or rising very slowly. The phenomenon of "second wind" is accompanied by a rise in diastolic pressure with disappearance of the high irregular systolic readings. In valvular disease of the heart with failing compensation, Dr. Mann finds a steady fall of diastolic blood-pressure with irregular maximum systolic readings. When a cardiac patient is allowed to get up for the first time a rise in the mean systolic pressure takes place. A fall in diastolic pressure should be taken as an indication that the patient has been allowed up too soon. Venesection in cases of advanced mitral failure to the extent of 10 to 20 ounces of blood was followed by a rise in the mean systolic pressure, and after about four hours a rise also in diastolic pressure was noticed. Strychnine and digitalis in cases of heart disease showed marked effects upon the arterial blood-pressure. In Bright's disease, acute and chronic, both systolic and diastolic pressure are enormously increased. On the other hand, the lowest readings recorded were obtained in a case of Addison's disease. Such are briefly some of the results of Dr. Mann's investigations. They are sufficient to suggest the usefulness of the manometer as an instrument for clinical investigation and observation.

---

NON-MALIGNANT tumours of the spermatic cord are comparatively rare, with the exception of cystic swellings. The diffuse hydrocele described by Pott has never, we believe, been met with since the days of that celebrated surgeon, and hitherto the case of fibroma of the cord recorded by Ferguson in 1856 has been considered one of those rare pathological curiosities often talked of and never seen. It is true other observers have recorded well-authenticated cases of fibroma of the cord, among the number being Poorson and Andre, Lenoir, Satriano and Parona, but the condition is still such a rarity that the record of a new case cannot fail to be of interest. Drs. Dubar and Leroy, of the surgical clinic at Lille, are the recorders, the patient being a mason, aged 32 years, who was admitted to the *Charité* for the radical cure of inguinal hernia. Since four years of age he had worn a right-sided truss. At the operation a small hernial sac was found behind the cord. A small oval tumour " the size of a nut " was found attached to the cord. This, on examination, proved to be a fibroma, subsequent microscopical examination showing " an area of degenerating hyaline material analogous to that which occurs in uterine fibroids." Only ten cases of this rare condition, say the authors, are on record. Clincally, they are met with in middle-aged patients, as slow-growing tumours, generally scrotal in situation. They are usually painless, but may cause neuralgia by pressure. In two recorded cases they were bilateral. They may give rise to mistakes in diagnosis, and really cannot be accurately diagnosed before operation. Their treatment is by excision, an operation which, in the recorded cases, proved an easy one.

---

HERRENKNECHT has recently published an interesting report of his experience with ethyl chloride anæsthesia. He has conducted three thousand anæsthesias with ethyl chloride without any mishap, and considers that when carefully used by those experienced in its administration it is the safest anæsthetic at our disposal, not excepting laughing-gas. The action of the anæsthetic may be divided into four stages: First, the prenarcotic analgesic stage; second, the stage of excitement; third, the stage of deep sleep; and fourth, the post-narcotic analgesic stage. If the patient shows signs of awakening before the surgical procedure has been finished, a second anæsthesia may immediately be begun, but in general for operations that are likely to consume more than five minutes it is preferable to begin with ethyl chloride and continue with ether or chloroform. Operations requiring only a half to one minute may be performed in the prenarcotic stage. During this the patient is conscious, but has little or no sensibility to pain. Healthy persons are able to walk home alone immediately after the operation. An important observation is that during the anæsthesia erotic delusions are often present, so that especially in dealing with women it is desirable to have witnesses at hand. The author uses an Esmarch chloroform mask with an impermeable covering leaving an empty space between the covering and the flannel. Through a small circular opening cut in the covering the anæsthetic is sprayed on the flannel.

---

THE treatment of diabetes in private practice is often one of the most difficult problems that the practitioner has to face, and all indications that can be of help will merit careful attention. The value of opium and its derivatives in the treatment of glycosuria is too well known to need comment, but in many cases it is found that these excellent drugs hardly affect a particular patient under observation. One of the latest substitutes for the opium treatment is santonin, which Journet has recently investigated. This author states that under the santonin treatment the results are in many cases quite astonishing. The patient's strength is increased, the polyuria is lessened, the thirst and dryness of the mouth are bettered, and the glycosuria rapidly diminishes. The drug is prescribed in powder or pill form, and in somewhat large doses, three to six grains (three to six lozenges of the pharmacopœial preparation) being given thrice daily. The patient is watched carefully and the drug is stopped after two days, being resumed at the end of a similar period.

---

RUDAUX, in a clinical lecture, gives a summary of the indications for rupture of the membranes which will be of service to every practitioner. So long as the contractions are normal and dilatation incomplete, the membranes should be kept intact. When the uterus is abnormally developed by excess of amniotic fluid, by multiple pregnancies or by a very large fœtus, primary uterine inertia may come on, in which case rupture of the membranes may stimulate the uterus to contract. Early rupture is advisable when the presentation has a tendency towards displacement, and is liable to become transverse; also after external version, when it is difficult to keep the child in the desired position. Presentations of the cord are better treated when the membranes are ruptured before reposition is attempted. The most urgent indication for rupture, however, is in hæmorrhage due to placenta prævia. A large rent made in the membranes will prevent the pull which these structures exert on the placenta, and the pressure of the descending part will stop the bleeding. Twin pregnancies often demand prompt rupture of the membranes.

DR. KATHERINE S. CLARKE, in a thesis on rheumatoid arthritis, concludes, from a study of a large number of cases, that there is strong evidence in support of the theory that the disease is of the nature of a cerebro-spinal toxæmia. The bacterial theory, that the joint troubles are due to specific organisms, is improbable because, although an immense amount of research work has been done with the object of demonstrating the presence of organisms which could be held to be specific, no uniformity has been obtained in the results of different observers. The increased pulse rate, without any concomitant organic cardiac lesion, which is a well recognised symptom present in the majority of cases, and the relapses and exacerbations which are frequently met with, the febrile attacks and gastro-intestinal disturbances, all favour the view that the disease is due to toxæmia. The pathological changes found at autopsies have been specially studied by Dr. Clarke. Definite pathological changes were demonstrable in the spleen, liver, ovaries, pancreas, stomach, kidney, and skin. In the majority of these there was marked increase in the connective tissues of the organs, with thickening and hyaline degeneration of the vessel walls. These changes were marked in the skin, and they can only be adequately explained "by the circulation of some chronic irritant in the blood-stream, which, in its course, involves joints as well as organs."

---

AT a recent meeting of the Edinburgh Medico-Chirurgical Society Professor Dowden read an interesting paper on "A Hundred Consecutive Operations for Appendicitis: Some Deductions Therefrom," in the course of which he laid stress on the many problems which the practitioner has to face in dealing with the acute form of this disease. Among the possible etiological factors in his series were carious teeth and a septic condition of the mouth generally, and one, at least, of his observations on the course of the disease cannot be too earnestly commended for consideration. "The administration of opium renders not only a definite diagnosis difficult, but masks the severity of the attack to a very serious extent. It cannot be too strongly emphasised that every attack of so-called acute or subacute "indigestion" calls for a most careful local examination, including, if there is doubt, a rectal examination as well." The mistakes in diagnosis recorded in the series are interesting. In three perforated gastric or duodenal ulcer was diagnosed; in another the case was sent to hospital with a diagnosis of suppurative arthritis of the hip joint, a large appendicular abscess of many days' duration, and extending below Poupart's ligament, being found on operation. As to treatment, Professor Dowden is emphatic on the subject of early operative interference. In cases where such interference is impracticable, morphia must be withheld and the patient put on starvation diet, with saline enemata and local application of heat or cold, whichever is found most comforting. Of the 100 cases, 82 were acute cases and 18 interval cases. The mortality in the latter was nil, in the former class it was 16.

DR. LERMOYEZ recommends the use of Fein's Adenotome, constructed on the crank principle for the operation of removing adenoids. He considers this instrument a great advance in the technique of adenotomy. The point of rotation is outside the mouth, while the range of action is more extensive than that in the ordinary curette. Dr. Lermoyez also speaks in favour of Camus' mask for the administration of accurate doses of ethyl chloride in the induction of anæsthesia for this operation. In common with English authorities Dr. Lermoyez has discontinued the use of ethyl bromide on account of its bad after effects.

---

IN a paper read before the American Laryngological, Rhinological, and Otological Society, Dr. John J. Kyle dealt with the ear symptoms associated with arteriosclerosis, especially between the ages of thirty and fifty years. Local or general arteriosclerosis gives rise to the following ear symptoms:—Tinnitus aurium (unilateral or bilateral), deafness (slight in degree, but progressive), loss of conduction through air and bone, dizziness, and occasionally auditory hallucinations. In the diagnosis of this condition, Menière's disease, the effects of drugs and other toxic substances, as well as functional deafness and labyrinthine hæmorrhages due to injury, must be excluded.

---

SIMONINI, in a recent number of *Il Morgagni*, reports the results of an extensive and systematic trial of the treatment of chorea by means of parathyroid extract. In two cases he found at the autopsies of children who had died of the disease that there existed extensive pathological changes in the parathyroid bodies, and it is known that the convulsive attacks in animals who have been deprived of their parathyroids can be arrested by the administration of extract prepared from the fresh glands. These facts led the author to try parathyroid extract in cases of chorea, and the results he has obtained are certainly encouraging enough to warrant a further trial of this method of treatment. Five cases were treated with perfect success, the patients recovering rapidly.

---

DECAPSULATION of the kidney as a method of treatment in eclampsia has scarcely had the attention it deserves since it was proposed by Edebohls. The many grave objections to the operation have been very closely discussed by most operators, and the success that has followed the relatively few attempts has not been encouraging. Last month, however, Falgowsku, of Munich, reported a successful case in which the operation was done on a woman, aged twenty-five, for post-partum eclampsia and acute nephritis. The eclamptic fits came on thirteen hours after confinement, and the patient was in a very grave condition twenty hours later, as all treatment was unsuccessful. Operation was decided upon, and both kidneys were decapsulated. The fits ceased, and six hours after the operation the patient was sleeping quietly. She made an excellent recovery.

